# An expanded set of genome-wide association studies of brain imaging phenotypes in UK Biobank

**DOI:** 10.1038/s41593-021-00826-4

**Published:** 2021-04-19

**Authors:** Stephen M Smith, Gwenaëlle Douaud, Winfield Chen, Taylor Hanayik, Fidel Alfaro-Almagro, Kevin Sharp, Lloyd T Elliott

**Affiliations:** 1 Wellcome Centre for Integrative Neuroimaging (WIN FMRIB) University of Oxford, United Kingdom; 2 Department of Statistics and Actuarial Science Simon Fraser University, Canada; 3 Genomics PLC, Oxford, United Kingdom

## Abstract

UK Biobank is a major prospective epidemiological study, including multimodal brain imaging, genetics and ongoing health outcomes. Previously, we published genome-wide associations of 3,144 brain imaging-derived phenotypes, with a discovery sample of 8,428 subjects. Here we present a new open resource of GWAS summary statistics, using the 2020 data release, almost tripling the discovery sample size. We now include the X chromosome, and new classes of image derived phenotypes (subcortical volumes and tissue contrast). Previously we had found 148 replicated clusters of associations between genetic variants and imaging phenotypes; here we find 692, including 12 on the X chromosome. We describe some of the newly found associations, focussing on the X chromosome and autosomal associations involving the new classes of imaging-derived phenotypes. Our novel associations implicate e.g. pathways involved in the rare X-linked syndrome STAR (syndactyly, telecanthus and anogenital and renal malformations), Alzheimer’s disease and mitochondrial disorders.

UK Biobank (UKB) is now approximately halfway through imaging 100,000 volunteers; the early-2020 release of brain imaging data contained data from almost 40,000 participants. This spans 6 brain MRI (magnetic resonance imaging) modalities, allowing the study of many different aspects of brain structure, function and connectivity. In conjunction with other data being recorded by UKB, which includes health outcomes, lifestyle, biophysical measures and genetics, UKB is a major resource for understanding the brain in human health and disease.

Previously, we presented genome-wide association studies (GWAS) of 3,144 brain imaging phenotypes, with a discovery sample of 8,428 subjects^1^. At that point we identified 148 replicated clusters of associations between genetic variants and the phenotypes. We found links between IDPs and genes involved in: iron transport and storage, extracellular matrix and epidermal growth factor, development, pathway signalling and plasticity.

We have now expanded and enhanced this work, with an almost threefold increase in sample size, an increase in the number of IDPs to almost 4,000, and with a focus on X chromosome associations carried out for the first time (including around 10 million variants with minor allele frequency ≥ 1%). The new classes of IDPs, computed on behalf of UKB and released for general access, are: subnuclei volumes in amygdala, brainstem, hippocampus and thalamus, Brodmann area FreeSurfer metrics and FreeSurfer-derived white-grey intensity contrasts. We have also greatly expanded our set of imaging confound variables^2^, reducing the likelihood of finding artefactual associations. GWAS summary statistics and Manhattan plots for all 3,935 phenotypes are freely available for download from the Oxford Brain Imaging Genetics (*BIG40*) web server (available at https://open.win.ox.ac.uk/ukbiobank/big40/), which also includes detailed tables of all IDPs, all SNPs (single nucleotide polymorphisms) tested, all association clusters, and an interactive viewer allowing for detailed interrogation of IDP associations with SNPs and nearby genes. We also provide a list of causal genetic variants for our top X chromosome clusters using a statistical fine-mapping approach^3^.

We conducted sex-specific GWAS on autosomes (chromosomes 1-22) and the X chromosome, followed by a meta-analyses combining these, using Fisher’s method. The X chromosome accounts for about 5% of the human genome and incorporates over 1200 genes, including many which play a role in human cognition and development. However, testing for association with genetic variants on chromosome X requires special consideration^4^. While genetic females inherit two copies of the X chromosome, genetic males inherit only a single copy from the maternal line (here we refer to people with two X chromosomes as genetic females, and people with one X and one Y chromosome as genetic males). The short pseudoautosomal regions (PAR) on the ends of chromosome X are homologous with parts of chromosome Y and can be analysed in the same way as autosomal chromosomes. For the non-pseudoautosomal region, a mechanism has evolved to balance allele dosage differences between the genetic sexes (X chromosome inactivation, or XCI); during female development, one copy is randomly inactivated in each cell. This means that maternally and paternally inherited alleles would be expected to be expressed in different cell populations within the body approximately 50% of the time. However, this dosage compensation mechanism (DC) is imperfect; it is currently thought that only 60-75% of X-linked genes have one copy completely silenced in this way^5^.

To account for this in GWAS, it is common to assume full dosage compensation^4^: males are treated as homozygous females with genotypes coded (0,2) according to whether they have 0 or 1 copy of the alternative allele. Joint analysis of genetic females and males with simulation studies^6^ suggest that type-I error control under this approach is reasonably robust to deviations from other assumptions (such as no sex-specific differences in allele frequencies), provided genetic sex is included as a covariate.

Recent studies have used the large sample sizes afforded by UKB to perform stratified analyses to estimate the degree of dosage compensation as a parameter across a broad variety of traits^5^. These studies suggest that only a small proportion of genes escape XCI, although the appropriate amount of DC shows considerable variation amongst traits. For educational attainment, Lee et al. ^5^ estimated a DC factor of 1.45, but concluded that little power was lost in their joint analysis irrespective of the assumed model.

While a joint analysis under a full DC model is a reasonable default, the power afforded by biobank-scale datasets also permits examination of possible sex-specific effects via stratified analyses. These stratified analyses can subsequently be meta-analysed; if the meta-analysis is based on estimated effects (regression beta values), appropriately chosen weights can give results almost the same as those from a joint genetic male/female analysis corresponding to any assumed DC model^5^. Nevertheless, results will be biased if the assumed DC model differs from the truth. An unweighted meta-analysis based on *p*-values using Fisher’s method (explored in our research), though potentially less powerful, should avoid this possible bias (as it is not sensitive to any relative scaling in the regression model and hence the effect sizes in the two sex-separated GWAS), and still have value in confirming signals from a joint analysis.

The Oxford Brain Imaging Genetics (*BIG40*) web server includes a summary statistics resource including results for our discovery cohort, and a full GWAS on all samples passing QC (with discovery and replication cohorts combined). A browsable interface is also provided for both of these subsets. In addition, the results of the sex-separated GWAS on chromosome X and autosomes are provided. We have also provided the summary statistics to the European Bioinformatics Institute (EBI) GWAS repository. More details about our resource are provided in Online Methods.

## Results

### Overview of GWAS Results

We conducted a genome-wide association study using the 39,691 brain imaged samples in UK Biobank. We divided these samples into a discovery (N=22,138) and a replication (N=11,086) cohort. The details for the imaging and genetics processing and the cohorts are given in Online Methods. We applied automated methods for identifying local peak associations for each phenotype, and also for aggregating peaks across phenotypes into clusters of association. A cluster is a set of phenotype/variant pairs such that all of the phenotype/variant pairs have a –Log10(*P*) value for association that exceeds a 7.5 genome-wide significance threshold, and such that all of the pairs have variants that are close with respect to genetic distance. We assigned each of the phenotype/variant pairs to one and only one cluster. We defined a cluster as replicating if at least one of the phenotype/variant pairs had nominal significance in our replication cohort (*p*-value < 0.05).

With these methods, we found 10,889 peak associations among all phenotypes and chromosomes (8,446 replicating at nominal significance), and found 1,282 clusters (692 replicating) after clustering the peak associations according to our automated methods (the number of replicating clusters reported in previous work^1^ was 148). The 692 replicating clusters are distributed across all chromosomes, with between 8 and 60 clusters per chromosome. We grouped the IDPs into 17 categories (Supplementary Table 1). Of the replicated associations among these 692 clusters, 16 out of 17 categories are represented (the *task fMRI activation* category is the only category without at least one association). The number of associations per category ranges between 12 for the category *volume of white matter hyperintensities (lesions)* which consists of just one IDP, and 1,954 for the *regional and tissue volume* category. All of these associations are listed in Supplementary Table 4, and Manhattan plots along with quantile plots are provided on the *BIG40* open web server.

**Figure 1:**
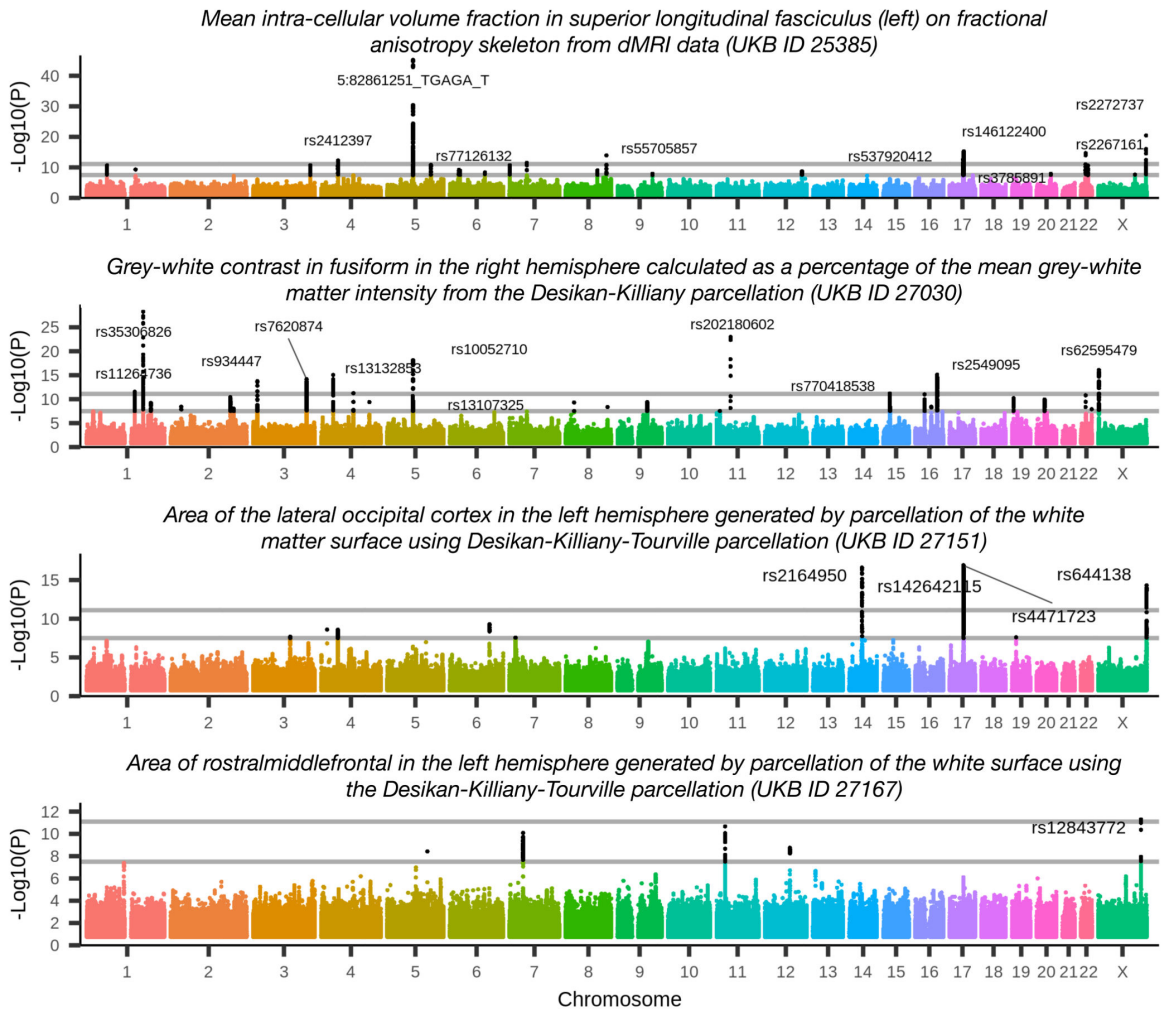
Manhattan plots for the four phenotypes achieving Bonferroni corrected significance on the X chromosome. Genetic variants are labelled for peak associations achieving the Bonferroni level. Plot titles indicate phenotype definition (including UKB ID field index from http://biobank.ctsu.ox.ac.uk/crystal/field.cgi?id=25385, 27030, 27151 or 27167). Black dots indicate associations that are significant associations at the genome-wide level, −Log10(*P*) ≥ **7.5**. Grey lines show genome-wide+Bonferroni level (11.1) and genome-wide significance level (7.5). These associations involve diffusion MRI and the Desikan-Killiany and the Dessikan-Killiany-Tourville parcellations of white matter and grey matter.

Of all of our clusters of associations, 38 are on the X chromosome (12 replicating), and 4 of the X chromosome clusters have a phenotype/variant pair with association significance exceeding the more stringent Bonferroni corrected level of –Log10(*P*) ≥ 11.1. (In this work we adjust for computing multiple GWAS by applying the Bonferroni correction on top of the standard GWAS threshold of –Log10(*P*) ≥ 7.5, resulting in a ‘Bonferroni’ threshold of 11.1.) These four clusters are investigated below, and Manhattan plots for the IDPs most associated with these clusters are displayed in Figure 1. We also investigate five novel clusters among the autosomal chromosomes.

We provide a fine-mapping of the four X chromosome clusters using the CAVIAR software^3^ with results described in Supplementary Table 3, considering regions within 250kbp of the lead associations for the clusters. For cluster 1, this resulted in a region containing 1,211 genetic variants. We ran the CAVIAR software^3^ with default settings and recorded the genetic variant found to be most causal for each phenotype association within the cluster. For cluster 1, CAVIAR reported rs2272737 (the same lead genetic variant found by our aggregation method) as the causal genetic variant for 81/97 of the phenotype associations. The situation was similar for the other three clusters; in each case, the lead genetic variant found by our aggregation method was among the genetic variants found to be causal by CAVIAR. (The number of genetic variants included in the 500kb regions examined by CAVIAR in the remaining three clusters included 2,017, 731 and 935 genetic variants, respectively. The proportion of phenotype associations for which the lead genetic variant found by our aggregation method was also estimated to be the most probable causal variant by CAVIAR for these remaining 3 clusters was 10/21, 5/35 and 5/17, respectively.) The CAVIAR results are detailed in Supplementary Table 3 and further details provided in the Supplementary Material.

**Figure 2:**
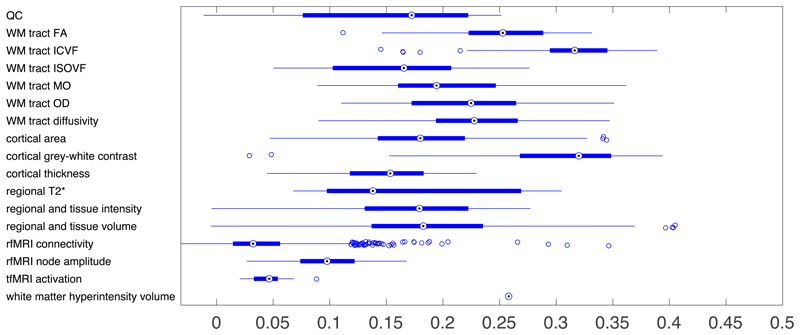
Heritability estimates (*h* ^2^) for phenotypes grouped according to IDP categories. Acronymns in *y*-labels include: quality control (QC), and diffusion tensor imaging phenotypes: white matter (WM), fractional anisotropy (FA), intra-cellular volume fraction (ICVF), isotropic or free water volume fraction (ISOVF), diffusion tensor mode (MO) and orientation dispersion index (OD). Boxplots indicate medians, 25th and 75th quantiles, and whiskers extending to maximal and minimal non-outliers (outliers are points exceeding 1.5 times the interquantile range from the median). More details for these 17 categories and heritability and standard errors for all phenotypes provided in Supplementary Table 1.

BIG40 also provides summary statistics for a GWAS with the discovery and replication cohorts combined (a *full-scan*). We also examined the heritability of each phenotype using linkage score regression^7^. The heritability for each of the phenotype categories is summarized in Figure 2. With phenotypes for which the estimated *h* ^2^ value was more than one standard error greater than 0, the estimated value of *h* ^2^ ranged from 0.01 to 0.41. The highest estimated heritability was found in phenotypes involved in regional and tissue volumes (as in previous work^1^), cortical grey-white contrast, and the intra-cellular volume fraction (ICVF) diffusion tensor imaging measure.

### X Chromosome Results - Overview and Sex-specific Tests

Full details for the lead associations for the X chromosome clusters (including clusters that do not replicate) are provided in Supplementary Table 2. A summary of all of the peak associations included in the X chromosome clusters is provided in Supplementary Table 3, and the full results for peak associations on all chromosomes are provided in Supplementary Table 4. In these tables, clusters numbers are given in the first column, and clusters are ordered based on the chromosome number (in ascending order with the X chromosome first) and then by the –Log10(*P*) value of the lead association (in descending order). A summary of all replicating X chromosome clusters is provided in Table 1, and further details including genes and expression quantitative trait loci (eQTL) for the four Bonferroni-significant clusters are provided in Table 2. Figure 1 shows Manhattan plots for the lead associations in these 4 top X chromosome clusters, and these clusters are explored further below.

**Extended Data Fig. 1:**
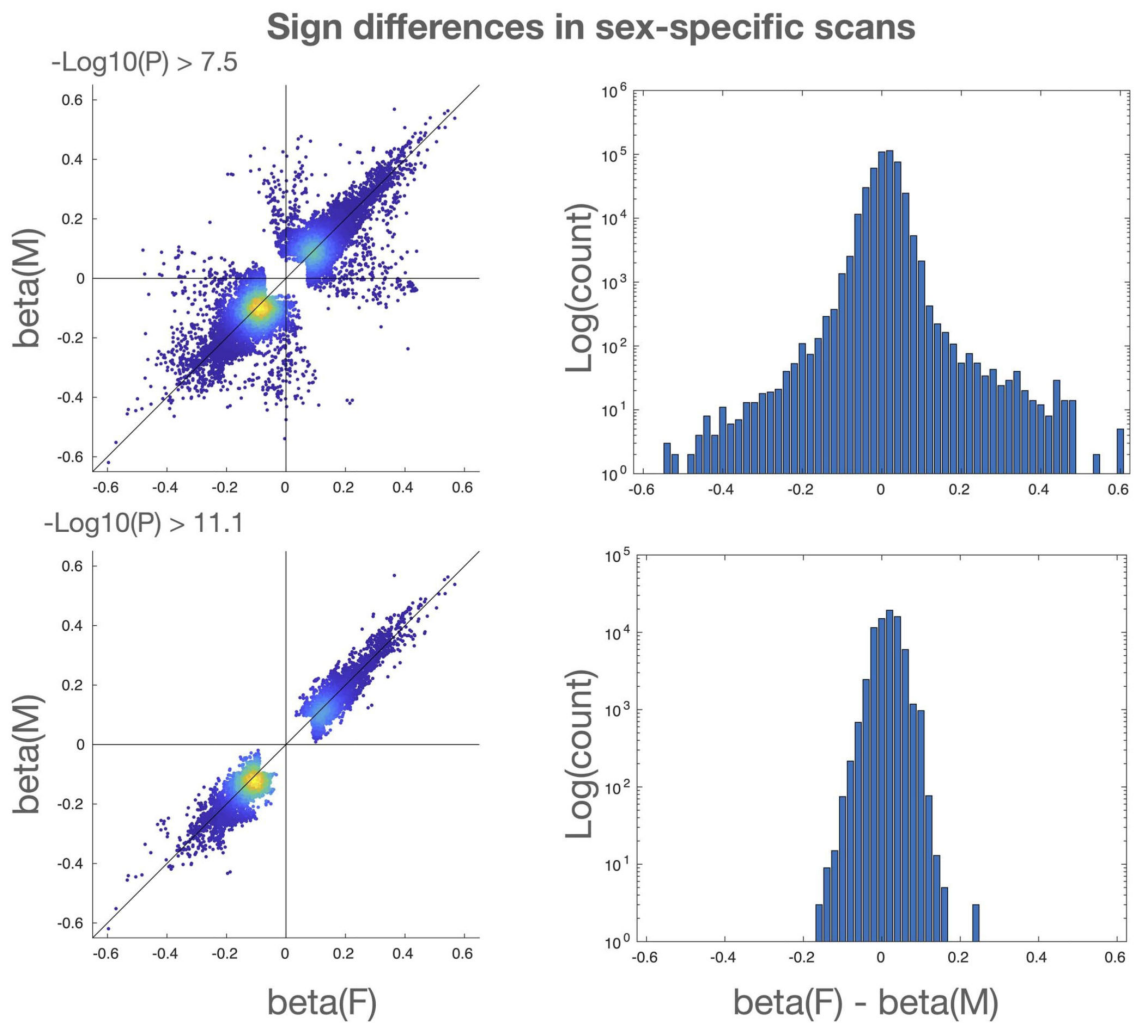
Comparisons of effect sizes and signs for genetic females and males Top row: Effect sizes for all associations with either genetic females or genetic males (or both) having -Log10(P) >= 7.5. Bottom row: effect sizes for all associations with either genetic females or genetic males (or both) having -Log10(P) >= 11.1. Left column: Scatter plots of effect sizes, indicating a small fraction (0.58%) of sign differences for -Log10(P) >= 7.5 and no sign differences (quadrants II and IV empty) for -Log10(P) >= 11.1 condition. Right column: Histograms of difference between effect sizes. Log y-scale indicates generally close matching of effect sizes.

Genetic sex affects the brain in fundamental ways^8^. Our main GWAS analyses include sex as one of the confound variables (in part, as sex is a causal factor in some strong imaging confound effects such as interaction of head size with image intensity and head motion). To assess the quality of this deconfounding, and to explore associations on the X chromosome that are driven by genetic sex, we conducted two additional GWAS in which we restricted our discovery cohort to just genetic females and (separately) just genetic males. We then combined these two additional GWAS in a meta-analysis using Fisher’s method. Clusters in our main analysis that are significant in the meta-analysis but not significant in one of the sex-specific scans may indicate sex-driven associations. Of the 12 replicating X chromosome clusters in the main analysis (with genetic male and female sexes combined in the discovery cohort), one cluster (Cluster 2) is significant at the 11.1 level for one genetic sex, but not significant for the other genetic sex; Cluster 2 may therefore be driven by genetic males. To provide more direct evidence for this, we performed two-tailed *z*-tests to determine if the beta coefficients differ significantly between the genetic sexes. For Clusters 1 and 2, we found that the beta coefficients are nominally different (Cluster 1: P=**1.2**×10^–2^, beta coefficient for genetic females: -0.14, beta coefficient for genetic males: -0.08; Cluster 2: P=**1.0**×10^–2^, beta coefficient for genetic females: 0.07, beta coefficient for genetic males: 0.13). The differences between the sex-specific beta coefficients for the lead associations of Clusters 3 and 4 were not significant. Among all of our associations (from all chromosomes and all IDPs) with at least one of the sex-specific scans significant at the –Log10(*P*) ≥ **11.1** level, the signs of the effect sizes for genetic females and genetic males always matched. For significance at –Log10(*P*) ≥ **7.5**, the signs matched 99.42% of the time (Extended Data Figure 1).

Finally, we created an additional set of clusters (using the clustering method described in the Supplementary Material), based on the *p*-values of the meta-analysis of the X chromosome (thresholding at the genomewide significance level). The clustering of the meta-analysis X chromosome scan produced 23 clusters. Twenty of these had lead associations within 0.25cM of one of the discovery cohort clusters derived from the original GWAS which pooled all subjects of both genetic sexes (indicating strong concordance between the meta-analysis and the discovery cohort). Each of the four discovery cohort X chromosome clusters with lead association at the Bonferroni level (the first four rows of Table 1) overlap with a meta-analysis cluster, suggesting that these main clusters are not confounded by genetic sex. Of the remaining meta-analysis clusters, three do not overlap with any of the discovery cohort clusters. The lead-rsid/lead-phenotype pairs of these three non-overlapping clusters are rs5990961/V3742, rs142994659/V1233 and rs764953454/V3919 (the mapping between the phenotype numbers and phenotype names is given in Supplementary Table 1). However, none of these three non-overlapping clusters achieved Bonferroni significance in the meta-analysis.

**Figure 3:**
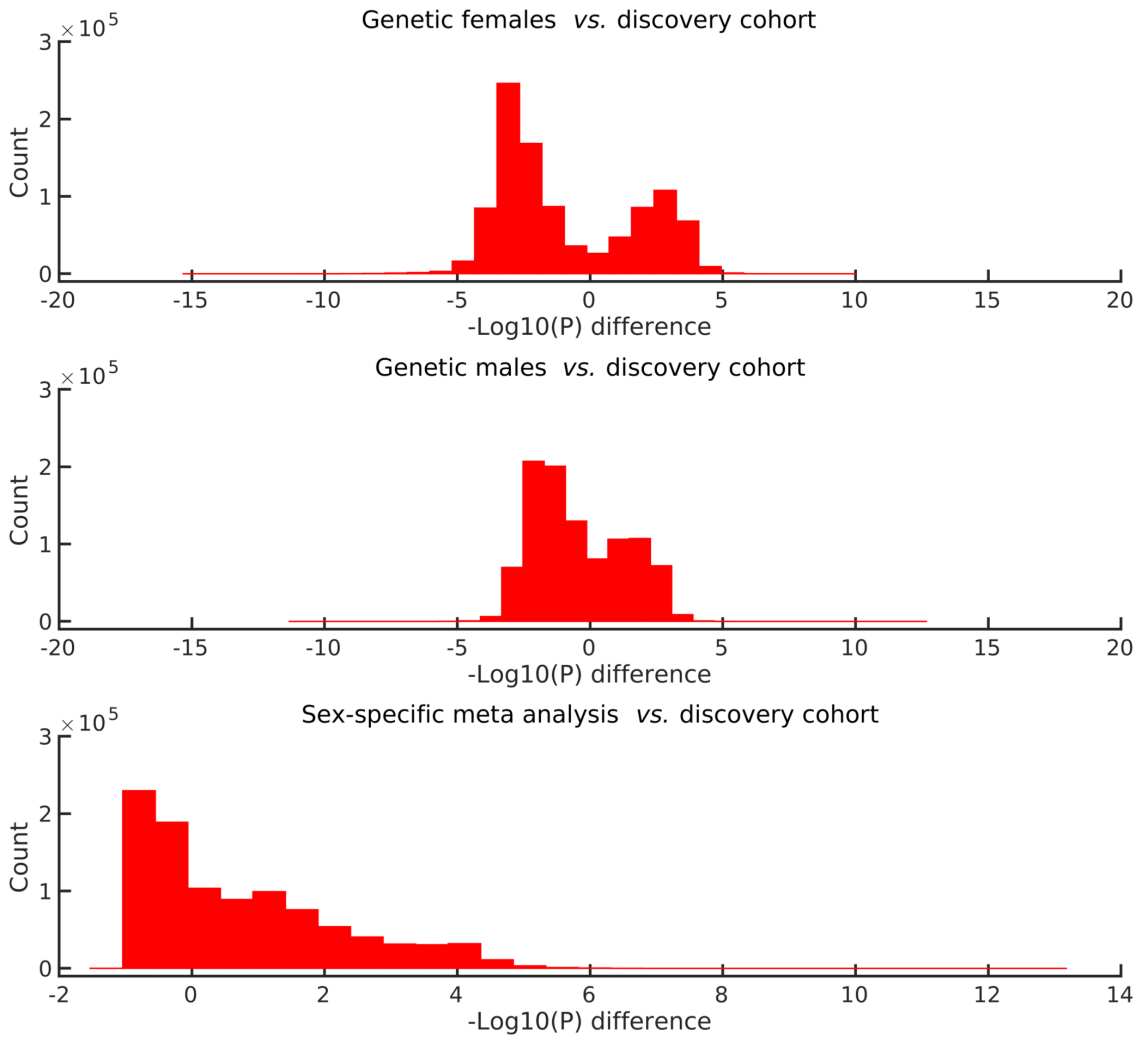
Paired difference histograms for the sex-specific scans on the X chromosome. We plot histograms for the differences between the −Log10(*P*) values for: genetic females (top), genetic males (middle), and the meta-analysis (bottom), vs. the discovery scan (which includes genetic male and female samples together, but did include a sex confound covariate). Differences are plotted for all associations for which the maximum −Log10(*P*) value over the four analyses is greater than 4.0, leading to the bimodal nature of the first two histograms. A total of 989,981 variants pass this maximum filter. The bottom plot shows that there is greater statistical sensitivity when carrying out sex-specific GWAS on the X chromosome, and then combining the results with a meta-analysis, than by combining all subjects together in a simple standard GWAS.

The differences in sensitivity between the main GWAS (pooling genetic male and female samples in the discovery cohort), the sex-specific GWAS and the Fisher meta-analysis are visualised concisely in Figure 3. The histograms show the distributions of paired-difference –Log10(*P*) values. For the sex-specific comparisons, there are SNP/phenotype pairs having reduced sensitivity compared with the original all-subjects GWAS (likely due to reduced statistical power because of reduced subject numbers), and other pairs with increased sensitivity (likely because a given association is stronger for the sex in question than for the other sex). The meta-analysis paired-difference distribution demonstrates that sex-separated GWAS followed by meta-analysis gives increased sensitivity to finding genetic associations in the X chromosome.

### Investigation of the Four Main X Chromosome Clusters

We now examine the four main X chromosome clusters in greater detail. The details for these additional investigations are summarized in Supplementary Table 5.

Cluster 1 comprises 5 SNPs, associated in total with 96 IDPs, all capturing differences in the properties of white matter tracts distributed throughout the cerebrum. This is described more in Table 2 and Supplementary Table 5. The top SNP (rs2272737, P=**3.5** × **10** ^–21^) is located about 10 kb away from, and is an eQTL of *FAM58A* (or *CCNQ*). The eQTLs reported throughout this work were assessed as significant according to the Genotype-Tissue Expression (GTEx) project. Mutations in this gene lead to STAR (syndactyly, telecanthus and anogenital and renal malformations), a rare X-linked developmental disorder recently identified^9^, for which notable brain variations have been observed such as incomplete hippocampal inversion, thin corpus callosum, ventriculomegaly and cerebellar hypoplasia^10^. In addition, while *FAM58A* codes for an orphan cyclin with undescribed function, it has been shown recently to interact with *CDK10* ^11^. Of particular relevance considering the many white matter IDPs associated with Cluster 1, mutation of the gene *CDK10* has been observed in a case study to lead to a rudimentary corpus callosum and paucity of white matter surrounding the lateral ventricles^11^.

The SNPs of Cluster 1 are further associated with an array of non-imaging-derived phenotypes (nIDPs—phenotypes not derived from MRI) largely related to health (including diagnosed diseases and operative procedures), as well as some variables not necessarily health-related (Supplementary Table 5). Interestingly, one SNP in Cluster 1 (rs1894299) was seen previously in a GWAS of Type 2 diabetes^12^. This SNP is located in an intron of *DUSP9* (*MKP4*), a gene that codes for a phosphatase whose overexpression specifically protects against stress-induced insulin resistance. This may be related to another consistent aspect of these nIDP associations with Cluster 1: the diet of UK Biobank participants with intake of sweet food and drinks (including desserts, puddings, beer and cider).

Cluster 2 comprises 9 SNPs associated altogether with 17 IDPs, all of which are grey matter *vs* white matter intensity contrast, in limbic and temporal regions, and brain areas making up the default-mode network^13^. The top SNP (rs62595479, P=8.2× 10^–17^) is located in a pseudoautosomal region of chromosome X, i.e., a genetic region homologous between chromosomes X and Y — in an intron of *DHRSX*, and is an eQTL of the same gene. The genetic association with the grey-white intensity contrast IDP for this SNP was mainly driven by the male UK participants (as described above). The male-dominated aspect of the association between *DHRSX* and the brain has also been observed in a study showing that four PAR genes, including *DHRSX* (and *SPRY3*, see below), are up-regulated in the blood of genetic male patients with ischemic stroke^14^.

While the majority of the nIDPs associations with the SNPs of Cluster 2 were related to diagnoses and operative procedures, half of these pointed at thyroid-related issues, in addition to the nIDP of workplace temperature, which may be related to thyroid function (Supplementary Table 5). Remarkably, the distribution of thyroid function modulation in the brain appears to consistently follow (in both positron emission tomography and fMRI studies), that of the 17 regions associated with Cluster 2: mainly limbic and temporal areas including the posterior cingulate cortex, orbitofrontal cortex, parahippocampal and fusiformgyrus^15^.

Cluster 3 includes 9 genetic variants associated with 28 IDPs of local brain volume. All 28 IDPs are located in the occipital lobe except for the volume of the brainstem and fourth ventricle. The top genetic variant (rs644138, P=4.8×10^–15^) is located in a PAR in an intron of *SPRY3*. This genetic variant is also an eQTL in many brain regions of a variety of genes whose mutations are involved in developmental and neurodevelopmental disorders: *RAB39B*, which plays a role in normal neuronal development and dendritic process, is associated with cognitive impairment^16^, X-linked intellectual disability^17^ and Waisman syndrome in particular, an X-linked neurologic disorder characterised by delayed psychomotor development, impaired intellectual development, and early-onset Parkinson’s disease^18^; *TMLHE* is associated with X-linked autism^19^; *CLIC2*, with X-linked intellectual disability^20^; and *BRCC3*, with an X-linked recessive syndromic form of moyamoya disease^21^. It is also an eQTL of *F8*, and *F8A1* (*DXS522E/HAP40*), a likely candidate for the aberrant nuclear localisation of mutant huntingtin in Huntington’s disease. Considering the distribution of the brain IDPs in the occipital lobe, it perhaps lends additional credence to the consistent, but yet not understood, observation of volumetric and sulcal differences in these visual grey matter regions in Huntington’s disease gene carriers^22^.

Another aspect of the genes for which the top genetic variant of Cluster 3 is an eQTL is cardiovascular issues: for instance, mutant *CLIC2* leads to atrial fibrillation, cardiomegaly and congestive heart failure^20^, *F8* encodes a large plasma glycoprotein that functions as a blood coagulation factor, whereas mutations in *BRCC3* are linked to moyamoya syndrome, a rare blood vessel disorder in which certain arteries in the brain are blocked or constricted, and that is accompanied by other symptoms including hypertension, dilated cardiomyopathy and premature coronary heart disease^21^. In line with this, we found that Cluster 3 was associated in UKB participants with diagnosis of atrioventricular block and ventricular premature depolarisation, as well as an operative procedure consisting of the replacement of two coronary arteries. In addition, Cluster 3 was consistently associated with many measures of physical growth, perhaps in line with the role of *SPRY3* as a fibroblast growth factor antagonist in vertebrate development, including height, lung function and capacity and body mass (Supplementary Table 5).

Finally, Cluster 4 comprised 8 genetic variants associated with volume of grey matter regions in the dorsolateral prefrontal cortex and the lateral parietal cortex (supramarginal gyrus and opercular cortex). The top genetic variant in this cluster (rs12843772, P=5.1 ×10^–12^) is located just <150 basepairs from *ZIC3*, which plays a key role in body pattern formation and left-right asymmetry. Mutations in this gene are thought to be involved in 1% of heterotaxy (situs ambiguous and inversus) in humans^23^. This may explain why the distribution of the higher-order grey matter regions associated with Cluster 4 follows the pattern of the fronto-parietal networks, which are notoriously left-vs-right segregated^24^. In particular, the supramarginal gyrus is known to show the strongest asymmetries from an early developmental stage^25^, and to be connected by white matter tracts that share a genetic influence with human handedness^26^. *ZIC3* is also involved in neural tube development and closure, and mutations in this gene cause, in addition to neural tube defects and cerebellum hypoplasia, consistent histological brain alterations with abnormal laterality and axial patterning, including a disorganised cerebral cortex^27^.

**Extended Data Fig. 2:**
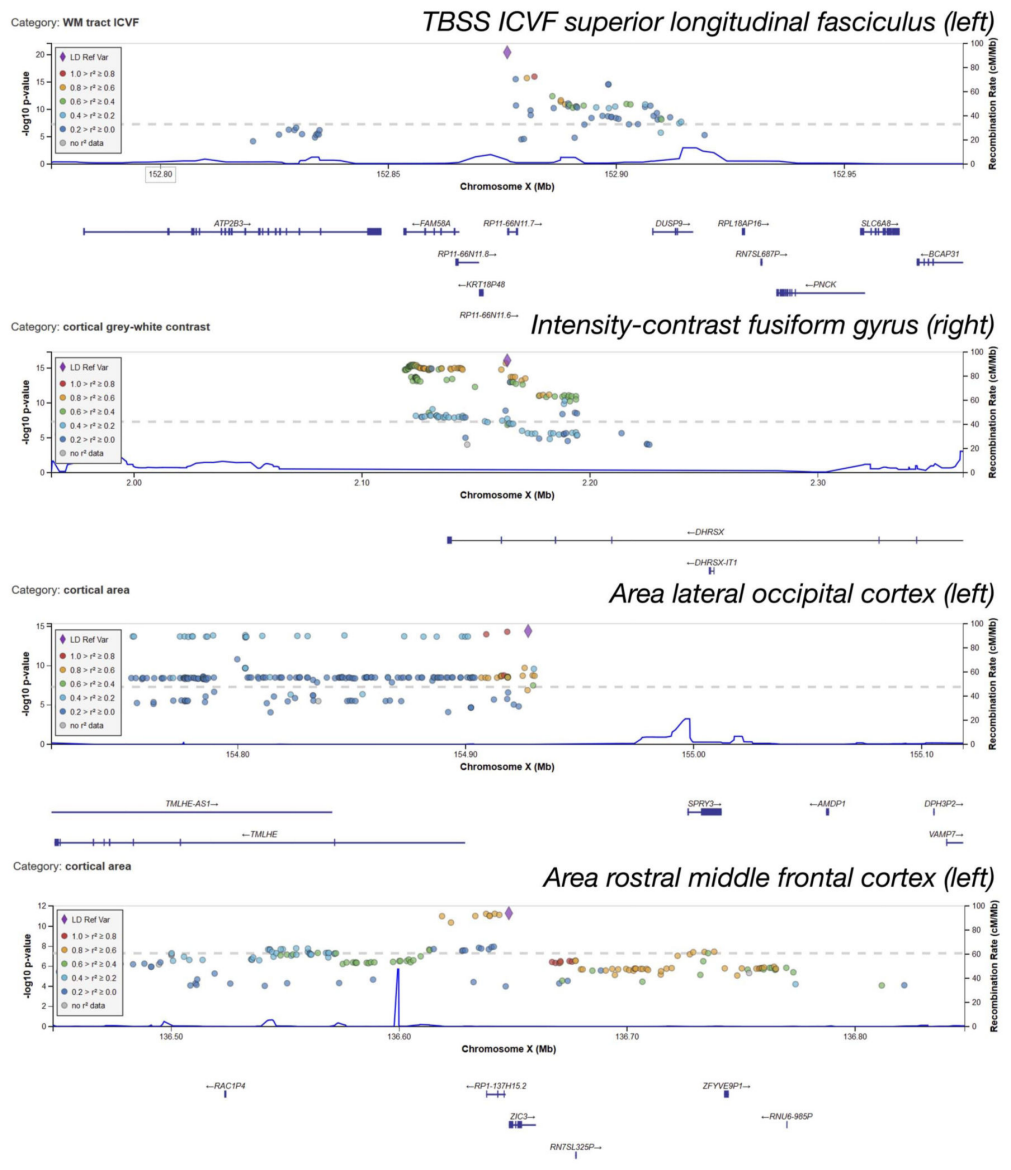
Regional association plots of the significant variants in X. First row: Region around rs2272737 (P = 3.5 × 10–21). This variant is an eQTL of FAM58A. Second row: Region around rs62595479 (P = 8.2 × 10–17). This variant is located in a pseudo autosomal region (PAR1) of X, in an intron of DHRSX. Third row: Region around rs644138 (P = 4.8 × 10–15). This variant is in an intron of SPRY3 (and is an eQTL in brain tissue of various genes). Bottom row: Region around rs12843772 (P = 5.1 × 10–12) located ≤150 bp from ZIC3. The genomic positions of the loci and genes are based on Human Genome build hg19. Regions considered include all loci within 10 kbp of the hit.

Cluster 4 is also in particular strongly associated with IGF-1 levels in the blood of UK Biobank participants (Supplementary Table 5). IGF-1 in particular controls brain development, plasticity and repair ^28^. More recently, it has emerged as a risk factor for dementia and particularly Alzheimer’s disease ^29^, as it is a major regulator of *Aβ* physiology, and controls *Aβ* clearance from the brain^30^. Zoomed-in views of these associations are displayed in Extended Data Figure 2.

With respect to XCI status of the genes discussed in this section, the gene *DHSRX* is located in PAR1 and is fully expressed in females^31^. Both *SPRY3* and *ZIC3* are subject to full inactivation^32^.

### Investigation of Five Autosomal Clusters

Among the 692 replicating clusters with GWAS significant *p*-values associations reported here, 48 involved a lead association among the autosomes with –Log10(*P*) ≥ **11.1**, replication (at nominal level), and which were distinct from the loci reported in previous work^1,33,34^ (*i.e*., more than 0.5cM from a reported genetic variant, and more than 1Mbp from a gene reported as associated in^33,34^). Of these 48 clusters, 7 involved associations with phenotypes from among the 3 new classes of UKB brain imaging phenotypes analyzed here. Of these 7 clusters, 2 (Cluster 271 with lead variant rs1368575 and Cluster 1070 with lead association rs3814883) were not related to previous brain imaging literature, and were not intergenic and were without eQTLs. The remaining 5 clusters (Clusters 163, 270, 841, 1029 and 1067 from the Supplementary Material Table 6) are investigated below. We provide a tabulation of all autosomal lead associations for our clusters with a cross-reference to^1,33,34^ in Supplementary Table 6.

The first of these most novel clusters related to previous brain imaging work (Cluster 163) included 12 distinct replicated variants associated with 31 IDPs, all but two related to the contrast between the intensity of the white matter and grey matter (tissue contrast: TC) generated by FreeSurfer, widespread across multiple cortical ROIs^35^. The top variant for this cluster (rs3832092, P=**1.9** × **10** ^–13^) is in an exon and an eQTL of *MARS2*, a mitochondrial methionyl-tRNA synthetase, and is associated in particular with parietal TC (precuneus, inferior and superior parietal cortex). Mutations of *MARS2* have been linked to spastic ataxia, as well as neurodevelopmental delay, and white matter abnormalities (leukoencephalopathy), cortical and cerebellar atrophy, as well as corpus callosum thinning^36^. This variant is further associated primarily with many nIDPs related to mass and body fat, as well as nIDPs involving allergy, sleep and red blood cell count and shape (according to the Open Targets Genetics platform).

The second cluster (Cluster 270) encompassed 13 replicated variants which were predominantly associated with the TC of 48 different cortical regions across the whole cerebrum. Its top variant (rs9290432, P=**2.4** × **10** ^–17^), associated mainly with temporal, parietal and prefrontal TC, is in an intron and eQTL of the gene *PLD1*. This gene codes for a phospholipid enzyme that has been shown to regulate the trafficking of the protein *β*APP^37^, the precursor of the amyloid beta whose plaques are a critical factor in the pathogenesis of Alzheimer’s disease. Increased expression of *PLD1* has been noted in the brains of Alzheimer’s disease patients both in the hippocampus and temporal lobe^38^, and *PLD1* is upregulated in the mitochondrial membrane from brains of patients with Alzheimer’s^39^. The top genetic variant of this cluster is in addition related to nIDPs of red blood cell count and shape (Open Targets Genetics).

The third cluster (Cluster 841) consisted of 8 replicated loci all associated solely with measures of TC in 15 mainly higher-order cortical areas. We find that the lead variant in this cluster (rs549893, P=**1.2** × **10** ^–12^), is associated with TC in the superior temporal cortex, and is located in an intron of *HMBS*, and is an eQTL of both *HMBS* and *VPS11* in many brain tissues (cortical, subcortical and cerebellar). The gene *HMBS* codes for an enzyme of the biosynthetic pathway of haem production. As such, mutations in *HMBS* are known to be related to acute intermittent porphyria, an autosomal dominant defect in the biosynthesis of haem. Acute intermittent porphyria manifests itself mainly as a neurological disorder, involving the autonomous, central and peripheral nervous systems, with acute life-threatening neurologic attacks. Deep cerebral white matter myelination abnormalities^40^, and axonal neuropathy^41^, have been observed in patients and animal models, as well as deficiencies in mitochondrial complexes in *Hmbs* mutant brain, suggesting that mitochondrial energetic failure also plays an important role in the expression of the disease^42^. In addition, mutations in *VPS11* in 5 patients have been suggested as leading to infantile onset leukoencephalopathy with brain white matter abnormalities, severe motor impairment, cortical blindness, intellectual disability, and seizures, as well as in a significant reduction in myelination following extensive neuronal death in the hindbrain and midbrain in an animal model^43^. The variant rs549893 is associated with nIDPs of body mass index, body fat mass and body fat percentage (Open Targets Genetics).

The fourth genetic cluster (Cluster 1029) comprised 17 replicated loci related again predominantly to TC in 45 widespread cortical areas. The lead variant for this cluster (rs140648465, P=**4.1** × **10** ^–16^) is associated with TC measures in the supramarginal, inferior and superior parietal cortex, and it is intergenic, and is an eQTL in cortical and subcortical tissues of *RMDN3/FAM82A2*, coding for a regulator of microtubule dynamics. This tether protein is involved in facilitating the lipid transfer by increasing the contact sites between the endoplasmic reticulum and mitochondria, particularly in the brain^44^. The protein FAM82A2 was found down-regulated in the frontal and parietal lobes in patients with Parkinson’s disease with dementia, with a dramatic reduction in the activity of the mitochondrial complexes^45^. Recently, reduced levels of the RMDN3 protein (sometimes also named PTPIP51) have also been found in the temporal cortex of the brains of Alzheimer’s disease patients, suggesting that a disruption of endoplasmic reticulum-mitochondria interactions mediated by *RMDN3* might be part of the neuropathological process in Alzheimer’s ^46^. A proxy genetic variant for the top variant of this fifth cluster (rs8042729, in LD of *R* ^2^=**1.00** with rs140648465) is further associated with nIDPs of blood count and hypertension (Open Targets Genetics).

The final interpretable cluster (Cluster 1067) comprised 31 replicated loci associated with a total of 51 IDPs of TC spread across higher-order cortical regions, as well as 19 other IDPs of cortical thickness (superior prefrontal and parietal), regional volumes (hippocampus, thalamus and choroid plexus) and white matter diffusion. This cluster’s most significant variant (rs2549095, P=**5.3** × **10** ^–20^) is associated with TC in an array for temporal, parietal and prefrontal cortical areas. This locus is in an exon of the gene *CLEC18A*, which codes for a C-type lectin that has only been recently characterized, and shown to be expressed in the brain, in microglia^47^. This locus is also part of a 40-panel of genes that make it possible to distinguish cardioembolic from large-vessel ischemic stroke with high accuracy^48^. A proxy locus for the top genetic variant (rs72785089, LD *R* ^2^=**0.81**) is further associated with measures of body mass and percentage, as well as blood count and alcohol intake frequency (Open Targets Genetics).

## Discussion

A major component in the expansion of the UK Biobank prospective epidemiological resource is the addition of tens of thousands of newly imaged participants, and the increase in the richness of phenotypes that can be derived from the imaging data. Since we published our first large-scale GWAS of UKB brain imaging in 2018, the brain imaging has almost reached its halfway point, having now scanned nearly 50,000 volunteers, of which 40,000 samples have already passed QC tests, and been processed and released for use in research. As a result, the size of the available discovery sample for GWAS has nearly tripled, and the number of genetic variants passing reliability thresholds (such as minor allele frequency MAF ≥ 1%) has increased by 30%, now reaching 10 million. It is now therefore a good time to update our large-scale GWAS, now with almost 4,000 imaging-derived phenotypes - thousands of distinct measures of brain structure, function, connectivity and microstructure. The number of replicated clusters of imaging-genetic associations identified has more than quadupled since our previous work. We have made all of the GWAS summary statistics openly available via the new *BIG40* brain imaging genetics server.

We also studied brain imaging associations in the X chromosome, and autosomal associations with novel phenotypes. We identified 4 X chromosome clusters that replicate at the GWAS+Bonferroni level (increasing the standard 7.5 level according to Bonferroni correction for the number of IDPs tested). Among these four top X chromosome clusters, we find associations involving diffusion measures distributed in white matter tracts and grey/white matter intensity contrasts. We also find associations involving occipital lobe grey matter, and fronto-parietal grey matter. These associations are relevant to a diverse set of variations in brain development and pathologies including the recently identified STAR condition (syndactyly, telecanthus and anogenital and renal malformations), Waisman syndrome, early-onset Parkinson’s disease, X-linked autism, Alzheimer’s disease and Huntington’s disease. Cardiovascular conditions such as ischemic stroke, moyamoya syndrome and premature coronary heart disease are also implicated. The X chromosome genes *FAM58A (CCNQ), DHRSX, SPRY3, F8A1, F8, BRCC3, TMLHE, RAB39B, CLIC2* and *ZIC3* are involved in the associations we report.

The X chromosome is typically understudied in GWAS, and many of the X chromosome loci we identify are now implicated in genetic associations with brain imaging phenotypes for the first time. Ploidy, the Barr body and potential confounding with genetic sex complicates X chromosome analysis. To address this, we performed sex-specific GWAS, and a meta-analysis combining the sex-separated analyses. We showed significant overlap between the sex-separated meta-analysis and the main GWAS (the discovery cohort pooling genetic females and males), providing evidence against confounding (and we note the significant sex-specific effect signs never differed for genetic females and males for variants with MAF ≥ 1%). This also allowed us to investigate if a given association was sex-driven (the association may be significant for only one genetic sex, or have significantly different effect sizes between the two sex-separated GWAS). For example, we found evidence that Cluster 2 for our brain-gene associations is driven by associations in genetic males.

Four of the autosomal clusters of association with grey-white tissue contrast were related to mitochondrial proteins and function. In the brain, mitochondrial disorders manifest themselves primarily as demyelinated lesions in the white matter, or as stroke-like lesions in both grey and white matter. More rarely, they present with cortical and subcortical necrosis^49^. While TC shows strong changes with ageing in prefrontal, lateral parietal, superior temporal and precuneus regions—those overall demonstrating the most significant associations with genetic variants—it is unclear what underlying tissue properties might be driving these effects in the MRI signal ratio^35^. Here TC, being almost entirely the only type of IDP showing significant genome-wide associations with our mitochondrial-related genetic clusters, appears to be specific and sensitive to mitochondrial function. These genetic-imaging findings are likely in line with the recent discovery that mitochondrial dysfunction, which emerges early during the ageing process, might prompt the catabolism of the myelin lipids of the white matter as an adaptive response to address brain fuel and energy demand^50^. This catabolic process perhaps explains the results previously observed with TC in ageing^35^.

Enhancing our understanding of the mapping between genotype and phenotype may lead to advances in neuroscience and improvements in outcomes for brain pathologies. Deeply phenotyped resources such as UK Biobank provide an opportunity to update the known genotype/phenotype mappings for a wide spectrum of brain imaging phenotypes as more samples and more phenotypes are released. It is crucial that such updates are provided on open platforms, accelerating the potential impact of brain imaging genetics. We have done that here with the open *BIG40* resource (with a total of 1,282 clusters of associations), and with freely available summary statistics. We hope that this dissemination will be valuable for the next generation of neuroscience research.

## Methods

We describe the image processing and genetic preprocessing and the association studies, including the procedures that are X chromosome specific, and the details of our sex-specific analyses. Specific details about our protocols are provided in the Life Sciences Reporting Summary.

### Image Processing

We used brain IDPs from the “40k” (approximately 40,000 participants) UK Biobank data release in early 2020, as processed by WIN-FMRIB on behalf of UKB^51^. After removal of subjects as part of the genetic processing (see below), we used data from 33,224 subjects. These were then randomly split into a discovery sample of 22,138 subjects (11,624 genetic females) and a replication sample of 11,086 subjects (5,787 genetic females). The ages in the discovery sample were: Females: mean age = **63.6** ± **7.3** years, min **= 45.1**, max **= 81.8**. Males: mean **= 65.0** ± **7.6**, min **= 46.1**, max **= 81.8**. In the replication sample: Females: mean **= 63.7** ± **7.4**, min **= 46.3**, max **= 81.6**. Males mean **= 65.0** ± **7.6**, min **= 46.1**, max **= 81.0**. The exact numbers of subjects vary across IDPs, according to patterns of missing data, with the maximum numbers given above (for IDPs with no missing data), and the minimum numbers being just 16% lower. The BIG40 online table listing the IDPs includes the exact number of subjects (in discovery, replication samples and in the sex-specific GWAS) for each IDP. The details for these IDPs (including long descriptions, category names and units) are summarized in Supplementary Table 1. The sample sizes we use represent the largest-to-date sizes released by the UK Biobank. No statistical methods were used to determine these sample sizes.

As described in detail in^52^, the UK Biobank data includes 6 MRI modalities: T1-weighted and T2-weighted-FLAIR (Fluid-Attenuated Inversion Recovery) structural images, susceptibility-weighted MRI (swMRI), diffusion MRI (dMRI), task functional MRI (tfMRI) and resting-state functional MRI (rfMRI). We (and colleagues)have developed and applied an automated image processing pipeline on behalf of UK Biobank^51^, https://www.fmrib.ox.ac.uk/ukbiobank/fbp. This removes artefacts and renders images comparable across modalities and participants; it also generates thousands of image-derived phenotypes (IDPs): distinct measures of brain structure and function.

In this work we used the 3,913 IDPs available from UK Biobank, spanninga range of structural, diffusion and fMRI summary measures (described in the central UK Biobank brain imaging documentation http://biobank.ctsu.ox.ac.uk/showcase/showcase/docs/brain_mri.pdf and listed in full on the BIG40 server https://open.win.ox.ac.uk/ukbiobank/big40/).

We also used 16 QC measures available from UKB, as well as 6 compact summary functional connectivity features (derived from the hundreds of individual connectivity features^1^). In this paper we refer to all of the above 3,935 measures together as “the IDPs.” Each IDP’s *Nsubjects ×* 1 data vector had outliers removed (set to missing, with outliers determined by being greater than 6 times the median absolute deviation from the median). We discarded subjects where 50 or more IDPs were missing (for any reason, which could be due to: data acquisition incompleteness; data quality problems as described in^51^, or the above-described outlier removal).

The data was then split into discovery and replication samples, and the remaining steps below applied to each sample separately. Each IDP’s data vector was quantile normalised^52^, resulting in it being Gaussian-distributed, with mean zero, standard deviation one. Confounds were removed from the data, in a manner similar to that carried out in^1^, including the 40 population genetic principal components supplied by UK Biobank and with a greatly expanded new set of confounds^2^. This includes confounds for: age, head size, sex, head motion during functional MRI, scanner table position, imaging centre and scan-date-related slow drifts. In order to maximise GWAS interpretability, we regress out all confounds listed and recommended in^2^. For higher-order (nonlinear and interaction) confounds, we used the same set of thresholds for automatic selection of these higher-order confounds. Given the slightly different set of subjects used here (for example, enforcing overlap with the genetics data) compared with those in^2^, this resulted in the 602 ‘maximal’ set of confounds (reported in^2^) being reduced here to 597 confounds.

### Genetic Associations

We consider the 488,377 samples included in the Spring 2018 release of the UK Biobank, and proceed with a preprocessing and discovery/reproduction paradigm similar to that described in^1^. Of the samples, 39,944 are included among the 41,016 samples in the UK Biobank for which IDPs are available, after the genotyping quality control procedures for sample removal specified in^53^. We removed samples without recent UK ancestry as determined by the *in.white.British.ancestry.subset* variable in the file *ukb_sqc_v2.txt* provided in the meta-data for UK Biobank. This variable selects samples based on self reported ancestry and genetic principal component thresholds. We also remove subjects based on relatedness, forming a maximal unrelated subset using the procedures recommended in^53^. This results in a maximally unrelated subset of 34,298 samples with recent UK ancestry and accepted genotyping and imaging quality control. We divided this set into a discovery and reproduction cohort according to a random 2/3 and 1/3 (respective) proportion, resulting in a discovery cohort of 22,865 samples and a reproduction cohort of 11,433 samples.

For the X chromosome, fewer samples were available after exclusions due to variation in karyotype or additional aspects of genotype quality control described in ^53^. This resulted in discovery cohorts of 22,853 (instead of 22,865 as listed above) for the non-pseudoautosomal region and 22,844 for the pseudoautosomal regions, and replication cohorts of 11,430 for the non-pseudoautosomal region and 11,426 for the pseudoautosomal regions (X chromosome aneuploidy was assessed using the *putative.sex.chromosome.aneuploidy* column of the genetic quality control file released by UKB^53^).

Next, we consider quality control filters for the minor allele frequency (MAF), information score (INFO^54^) and Hardy-Weinberg equilibrium (HWE). We apply filters for MAF ≥ 0.001 and INFO ≥ 0.3 and HWE –Log10(*P*) ≤ 7. For the X chromosome, we apply these filters using the *qctool* software version 2.0.1 and the *-infer-ploidy-from sex* flag. This implies that genetic males contribute half as much as genetic females towards MAF and INFO for the non-pseudoautosomal region of the X chromosome. Further, as has been recommended ^55^, for the X chromosome the HWE filters are applied after computing for genetic females only. After these filters, of the 93,095,645 genetic variants included in UK Biobank in chromosomes 1:22, 16,445,196 remain. And of the 3,963,707 genetic variants in the X chromosome, 657,883 remain (639,835 in the non-pseudoautosomal region and 18,048 in the pseudoautosomal regions).

### Genome-wide Association Studies

We performed linear association tests on the samples in the discovery cohort. We performed these tests between each of the 17,103,079 genetic variants and each of the 3,935 IDPs described above (10,119,893 of which have MAF ≥ 0.01). The genotypes were provided by UKB, and details for imputation (including X chromosome imputation) and genetic principal component construction are provided in^53^. We used the *bgenie* software^53^ to conduct the GWAS and record the effect sizes (beta), standard errors and –Log10(*P*) values for the associations. The effect sizes were recorded *in the direction of the alternate allele*. The phenotypes were scaled to have unit variance after deconfounding. The variants on chromosomes 1:22 were not scaled. Therefore, an effect size of 1.0 indicates that each copy of the alternate allele generally confers an increase in the phenotype by one standard deviation. For the non-pseudoautosomal region of X, the dosages for genetic males were scaled by a factor of 2.0 so that they lie in the range [0, 2], respecting the Barr body.

We produced Manhattan plots for each of the 3,935 IDPs, plotting the –Log10(*P*) value for each variant (these Manhattan plots are provided on the BIG40 open web server, along with quantile plots). For the Manhattan plots, we applied an additional filter of MAF ≥ 0.01 to all variants. A method for extracting hits (peak associations) from a genome-wide association study was developed in^1^. We applied that method to extract hits from the 3,935 scans with –Log10(*P*) values exceeding the GWAS threshold of 7.5 (again, with the MAF ≥ 0.01 filter), and annotated the Manhattan plots with these hits (note that in our tables we also signified results passing a Bonferonni corrected threshold for *p*-values below 1.0 × 10^–7.5^ divided by the number of IDPs: ~11.1). For each of the hits, we conducted a replication analysis by performing a linear association test on the samples in the replication cohort and record the effect sizes, standard errors and –Log10(*P*) values for the replication. We extended the method from^1^ in order to automatically generate clusters. A cluster is a set of phenotype/variant pairs such that each variant in the cluster is a peak association for its corresponding phenotype, and such that all variants are within a 0.25cM (centimorgans) distance of the phenotype/variant pair with highest –Log10(*P*) value in the cluster. Each phenotype/variant pair identified as a hit appears in one and only one cluster. The details of this clustering method are provided in Online Methods. We provide a software package called *Peaks* implementing these clustering methods, and have released it under the open source BSD 2-clause license.

Other methods for extracting lead associations include Bayesian methods such as CAVIAR^3^ for determination of causal genetic variants. We examined causal genetic variants in the top four X chromosome clusters by computing the linkage disequilibrium matrix using *plink2* ^56^ for all variants within 250kbp from the lead associations (our CAVIAR results are provided in Table 3 of this Supplementary Material and the results are summarized in the main text). Summary statistics for the associations of all genetic variants (with MAF ≥ 0.001) for the discovery cohort (as well as the sex-separated and meta-analyses GWAS, and a pooled discovery+replication GWAS - see below), are available for download on the BIG40 open web server.

### Full-Scan

We also provide for download on the BIG40 open web server the summary statistics for a version of this genome-wide association study conducted on the union of the discovery and replication cohorts considered in this study (i.e., a maximal subset of unrelated samples with recent UK ancestry, among *all* samples in the UKB 2020 release of approximately 40,000 brain imaged samples). The genetic variants considered in this scan are the same genetic variants passing our filters for the discovery cohort reported on in this paper (with MAF ≥ 0.001). The sample size of each association test (after considering missing phenotypes and X chromosome exclusions due to aneuploidy) are also provided (the maximum number of included samples over all phenotypes in this full scan is 33,224).

Using the summary statistics from this full-scan, we estimate the heritability of each phenotype. We used linkage disequilibrium score regression^7^ to produce these estimates. Linkage disequilibrium scores were sourced from the European population of the 1000 Genomes project^57^. Results are listed in Supplementary Table 1.

### Sex-specific Scans

We also considered a scan for association on genetic male samples only, and also a scan for association on genetic females only (in both cases, with samples from the discovery cohort). For these scans, in the non-pseudoautosomal X chromosome region, 11,885 genetic female samples were used, and 10,968 genetic male samples were used. For the pseudoautosomal regions, 11,882 genetic female samples were used and 10,962 genetic male samples were used. As in the main analysis, the number of samples per phenotype varies due to missingness (the sample sizes for each phenotype for genetic females and males are provided in Supplementary Table 1). We conducted these two scans for association between each deconfounded phenotype and each variant on the X chromosome and the autosome, and recorded the effect sizes (beta), standard errors and –Log10(*P*) values. We then combined these two scans in a meta-analysis using Fisher’s method^58^, providing a –Log10(*P*) value which is more strongly controlled for sex-specific effects. The equation for the combined *p*-value under this meta-analysis method is as follows:

(1) $$1-f_{X^{2}}\left(-2\left(\text{log}\;p_{m}+\text{log}\;p_{f}\right),4\right)$$

Here *f*_*X*^2^_(·, *v*) is the cumulative distribution function of a chi-squared random variable with *v* degrees of freedom, and *p_f_* and *p_m_* are the *p*-values of the genetic female and genetic male scans, respectively.

### The *Peaks* algorithms

In genome-wide association studies with thousands of phenotypes, we must determine when two peak associations for two different phenotypes are related, in the sense that the peak variants are close together in genetic distance and/or linkage disequilibrium. For studies in which the number of phenotypes (or strength and complexity of genetic associations) amounts to only a few supra-threshold results, such matching can be done by hand by examining recombination maps and linkage-disequilibrium diagrams. Such by-hand work is not feasible for studies with thousands of phenotypes. In this work, we provide a new automated method to coregister peak associations across many phenotypes. We provide a software package *Peaks* which implements these methods. The *Peaks* software provide an implementation of the algorithm in^1^ (in the *Identifying associated genetic loci* subsection of the Methods section) for uncovering peak associations for each phenotype, and an extension to co-localization of hits across phenotypes.

To combine peak associations into clusters that span phenotypes, we use a ‘greedy’ algorithm which delivers an optimally efficient clustering of genetic variant/phenotype pairs. The algorithm works by first identifying the peak associations for each phenotype (using the algorithm described in the previous subsection). Then, by iteratively extracting the genetic variant/phenotype pair with the top –Log10(*P*), and then assigning all lead associations for all phenotypes within 0.25cM of that genetic variant to the same cluster. The details are as follows:

1. For each chromosome, convert the chromosome’s peak associations into an array.
2. For each chromosome, convert the chromosome’s array into a binary max-heap keyed on the –Log10(*P*) of each genotype/phenotype pair, using the *O*(*n*) running time *heapify* algorithm described in^59^.
3. For each chromosome, while the chromosome’s heap is not empty, extract the maximum genetic variant/phenotype pair from the heap to create a new cluster. This can be done in *O*(Log(*n*)) time. This extracted pair is the lead association of the cluster. Then, the 0.25 cM-cover is removed from the chromosome’s heap, which is also an *O*(Log(*n*)) operation. The cluster is outputted (including the removed aspects).
4. Since there are at most *n* extractions and deletions the total running time is *O*(*n* Log(*n*)), which is optimal as it is tight to the sorting lower bound of *O*(*n* Log(*n*)), and since sorting is a relaxation of clustering and a lower bound of a relaxation has running time no worse than the original problem.

The open source *Peaks* software implementing these methods is available on *github* at https://github.com/wnfldchen/peaks.

## Extended Data

## Supplementary Material

Extended Data Figures 1 and 2

Supplementary Tables 1-6

## Figures and Tables

**Table 1:** Lead associations for the 12 replicating X chromosome clusters. Links to UKB phenotype definitions are provided in column 3. Summary statistics *beta* and *se* are provided in Supplementary Table 1. The −Log10(*P*) values provided are for the main discovery cohort. Column N_*SNPs*_ indicates the number of peak phenotype/variant pairs included in the cluster. Bolded rsids indicate significance at the Bonferroni threshold in the main discovery cohort.

#	rsid	Phenotype ID and brief description	Position	a1	a2	−Log10(P)	N_SNPs_
1	**rs2272737**	1943 White matter neurite density inleft superior longitudinal fasciculus	152876080	C	T	20.459	97
2	**rs62595479**	1408 White-grey T1 intensity contrast inright fusiform gyrus	2163736	T	C	16.088	21
3	**rs644138**	0818 Area of left lateral-occipital cortex	154927581	G	A	14.319	35
4	**rs12843772**	0834 Area of left rostral-middle-frontal cortex	136648126	C	T	11.292	17
5	rs188847578	0076 Volume of grey matter in left supplementary motor cortex	127661277	G	T	10.247	3
10	rs193203210	1437 Volume of white matter hyperintensiies	152634500	C	G	8.711	2
11	rs11539157	0202 Volume of left ventral diencephalon	68381264	C	A	8.659	1
15	rs149776026	3512 Functional connectivity, connection 1084 dimensionality 100	124604066	A	G	8.193	1
16	rs5916169	2510 Functional connectivity, connection 82 dimensionality 100	5507316	C	T	8.12	1
23	rs6527976	1437 Volume of white matter hyperintensiies	13808841	A	G	7.981	1
25	rs5930655	0136 Volume of grey matter in brain stem	133781599	A	C	7.925	1
30	rs6644158	0244 Volume of subiculum body in left hippocampus	113638468	G	A	7.723	1

**Table 2:** Context for the four X chromosome clusters with significance at the Bonferroni threshold. Cluster number (bolded and matching cluster numbering in the Supplementary Tables) and a general description of the phenotypes involved in the cluster are given in the first column. Cytogenetic positions are provided: clusters with both X and Y positions are in a pseudoautosomal region. Note that the cytogenic position Xq28 is on the edge of the non-PAR region and overlaps with the Y chromosome Yq12, although the lead association is in non-PAR. Information about expression quantitative trait loci (eQTLs) is provided. Column *sex* indicates if the sex-specific scan is significant at the Bonferroni threshold of −Log10(*P*) ≥ **11.1** for one genetic sex, but not significant for the other genetic sex (with *M* indicating the genetic sex for sex-driven effect in males, and dash indicating no such sex-driven effect).

Phenotypes for cluster	Gene	Position	eQTLs	eQTL tissues	Sex
**1**. Diffusion MRI measures in distributed white matter tracts	*FAM58A (CCNQ)* intergenic	Xq28	*FAM58A*	Brain - Cerebellum	-
**2**. Grey/white matter intensity contrast (temporal, limbic, default mode network)	*DHRSX* intron	Xp22.33/Yp11.2	*DHRSX*	Blood, artery, heart, musculo- skeletal	M
**3**. Occipital lobe grey matter area and volume, brainstem volume	*SPRY3* intron	Xq28	*F8A1, F8, BRCC3, TMLHE, RAB39B, CLIC2*	Brain - Cortex, subcortex, cerebellum	-
**4**. Fronto-parietal grey matter area and volume	*ZIC3* intergenic	Xq26.3	*RP11-158M9.1*	Brain - Cerebellum	-

## Data Availability

Our resource includes openly released summary statistics and results for a variety of GWAS paradigms on the most recent release of 3,935 UKB brain imaging phenotypes. These results are released on BIG40 (https://open.win.ox.ac.uk/ukbiobank/big40/), the European Bioinformatics Institute (EBI) and the supplementary material of this paper. An enumeration of the aspects of our resource is as follows: Summary statistics for our disovery cohort (*N* ≤); available on BIG40 (Manhattan plots, full downloads, and a browsable interface), EBI under study accession numbers GCST90002426-6360 (ftp://ftp.ebi.ac.uk/pub/databases/gwas/summary_statistics/GCST90002426 to ftp://ftp.ebi.ac.uk/pub/databases/gwas/summary_statistics/GCST90006360). Details of the clusters of associations identified by *Peaks*, including summary statistics for replication; available on BIG40 and in the Supplementary Tables 2 to 6. Causal variants identified by CAVIAR for the four X chromosome clusters significant at the –Log10(*P*) ≥ **11.1** level (Supplementary Table 5). A full GWAS on all phenotypes and chromosomes, with the discovery and replication cohorts combined (available on the BIG40 website as a download and as a browsable interface). Sex-specific GWAS on the discovery cohort with genetic females and genetic males considered separately, and combined through a Fisher meta-analysis (available as a download on BIG40). The heritability of each phenotype, assessed through LDSC on the full GWAS with discovery and replication cohorts combined (Supplementary Table 1).

## Code Availability

The following software packages and servers were used throughout this work:

- *bgenie* v1.3, software for efficient genome-wide association studies on high dimensional phenotype data. https://jmarchini.org/bgenie/ ^53^
- *qctool* v1.4 and v2.0.1, software for preprocessing genetic data. https://www.well.ox.ac.uk/~gav/qctool_v1/ and https://www.well.ox.ac.uk/~gav/qctool_v2/
- *Peaks* v1.0, novel software for extracting clusters from multi-phenotype genome-wide association studies. https://github.com/wnfldchen/peaks
- *PheWeb* v1.1.19, a webserver for browsing phenome-wide associations. https://github.com/statgen/pheweb ^60^
- *BIG40* open web server for Brain Imaging Genetics. https://open.win.ox.ac.uk/ukbiobank/pheweb/
- *plink2* v2.0, alpha software for conducting genome-wide association studies and preprocessing of genetic data https://www.cog-genomics.org/plink/2.0/ ^56^
- *CAVIAR* v2.0, fine-mapping software for extracting causal variants from summary statistics. http://genetics.cs.ucla.edu/caviar/ ^3^
- *Open Targets Platform* an online web server for genome-wide association study. https://genetics-app.netlify.app/ ^61^
- *LDSC* v1.0.1, software for heritability analysis from summary statistics (linkage score regression). https://github.com/bulik/ldsc/ ^7^
- The GTEx online resource. https://gtexportal.org/home/ ^62^
