# Supplementary material for: An expanded set of genome-wide association studies of brain imaging phenotypes in UK Biobank
Source: Nat Neurosci. Author manuscript; Available in PMC 2021 May 9. (PMC7610742; doi:10.1038/s41593-021-00826-4)
Supplement: Extended Data Figures 1 and 2 [file EMS123172-supplement-Extended_Data_Figures_1_and_2.pdf]

Extended Data Fig. 1: Comparisons of effect sizes and signs for genetic females and males

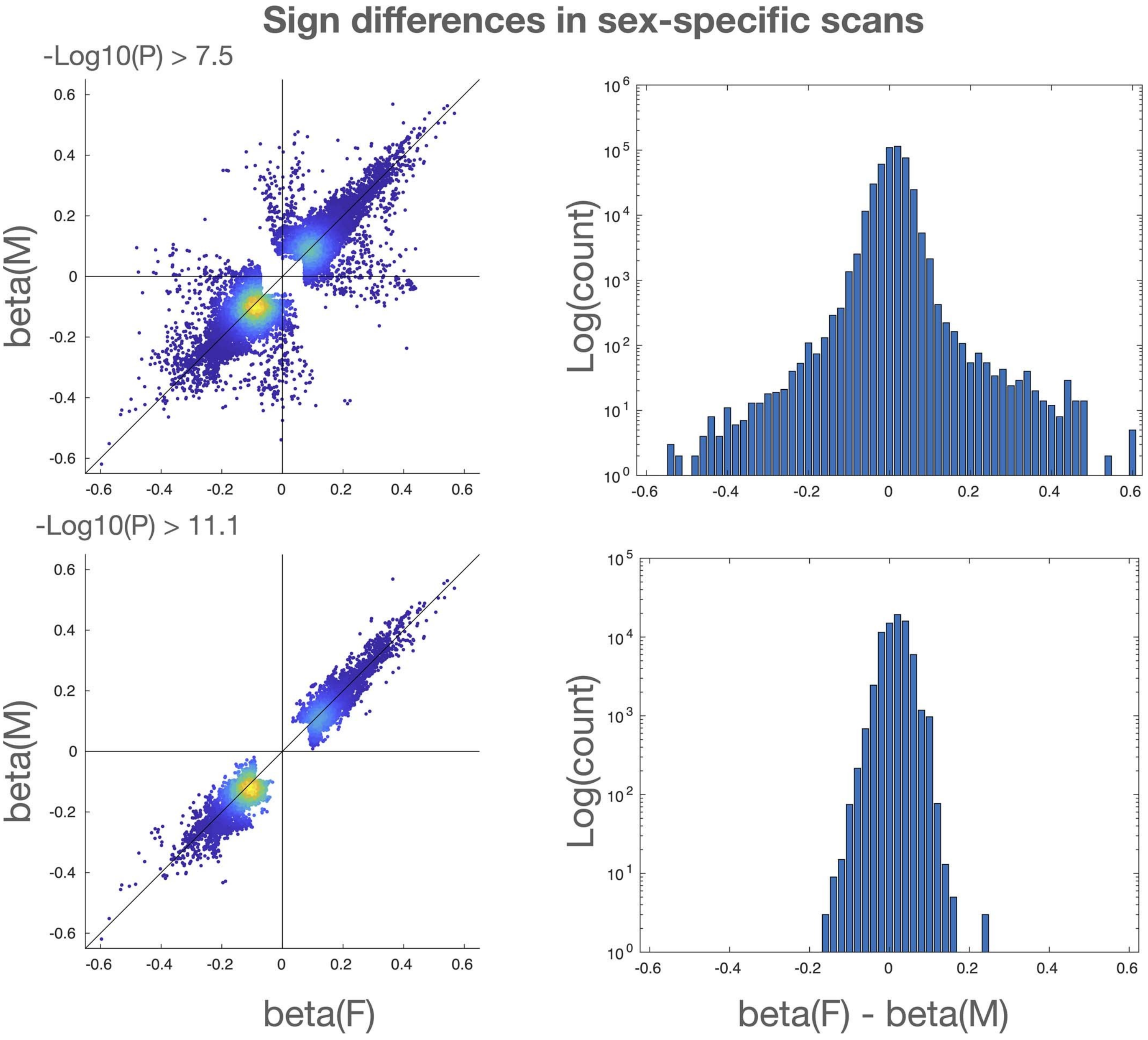

Top row: Effect sizes for all associations with either genetic females or genetic males (or both) having *-Log10(P) >= 7.5*. *Bottom row*: effect sizes for all associations with either genetic females or genetic males (or both) having *-Log10(P) >= 11.1*. *Left column*: Scatter plots of effect sizes, indicating a small fraction (0.58%) of sign differences for *-Log10(P) >= 7.5* and no sign differences (quadrants II and IV empty) for *-Log10(P) >= 11.1* condition. *Right column*: Histograms of difference between effect sizes. Log *y*-scale indicates generally close matching of effect sizes.

Extended Data Fig. 2: Regional association plots of the significant variants in X.

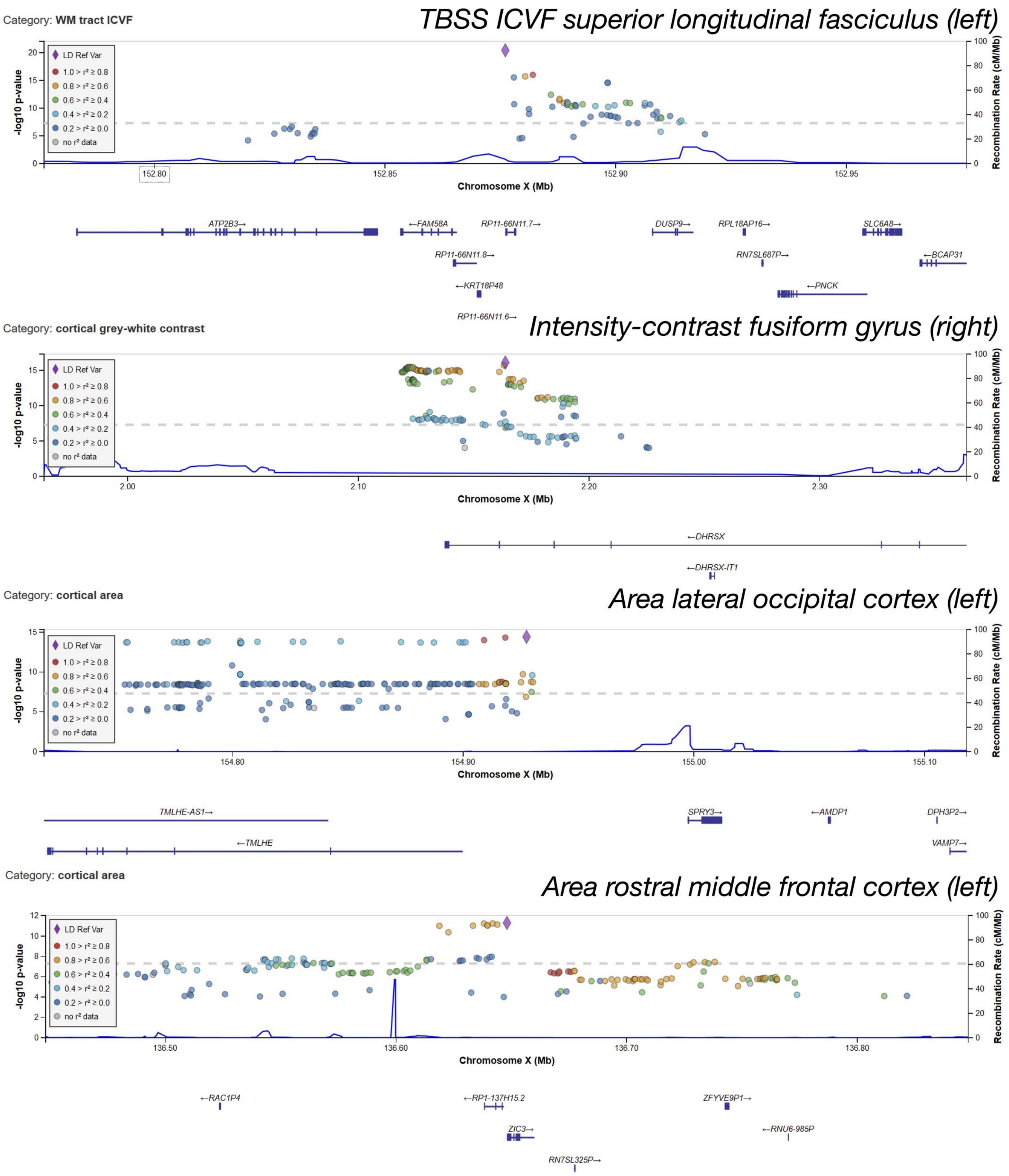

First row: Region around rs2272737 (P = 3.5 × 10−21). This variant is an eQTL of FAM58A. Second row: Region around rs62595479 (P = 8.2 × 10−17). This variant is located in a pseudo autosomal region (PAR1) of X, in an intron of DHRSX. Third row: Region around rs644138 (P = 4.8 × 10−15). This variant is in an intron of SPRY3 (and is an eQTL in brain tissue of various genes). Bottom row: Region around rs12843772 (P = 5.1 × 10−12) located ≤150 bp from ZIC3. The genomic positions of the loci and genes are based on Human Genome build hg19. Regions considered include all loci within 10 kbp of the hit.
